# Identical Strength of the T Cell Responses against E2, nsP1 and Capsid CHIKV Proteins in Recovered and Chronic Patients after the Epidemics of 2005-2006 in La Reunion Island

**DOI:** 10.1371/journal.pone.0084695

**Published:** 2013-12-23

**Authors:** Jean-Jacques Hoarau, Frederick Gay, Olivier Pellé, Assia Samri, Marie-Christine Jaffar-Bandjee, Philippe Gasque, Brigitte Autran

**Affiliations:** 1 Immunopathology and infectious diseases research grouping, GRI/IRG EA4517, University of La Reunion and Centre Hospitalier Universitaire (CHU North Felix-Guyon), Saint-Denis, Louisiana, United States of America Reunion - France; 2 Department of Medical Biology, Parasitology, Pitié-Salpêtrière Hospital, Pierre and Marie Curie University, Paris, France; 3 Laboratory of Immunity and Infection, Inserm U945, Paris, France; 4 Biology / Microbiology / Virology / Biochemistry Units, Centre Hospitalier Universitaire (CHU North Felix-Guyon) and University of La Reunion, Saint-Denis, Louisiana, United States of America Reunion - France; 5 Laboratory of Immunity and Infection, UPMC University Paris 06, Unité mixte de recherche-S945, Paris, France; 6 Inserm, IFR 113, Immunité-Cancer-Infection, Paris, France; Agency for Science, Technology and Research - Singapore Immunology Network, Singapore

## Abstract

To characterize the immunity developed by patients infected by chikungunya virus (CHIKV), we studied the intensity and specificity of CHIKV-specific T cells mediated responses in chronic and recovered patients at 12 to 24 months post-infection. T cells were challenged in vitro against CHIKV synthetic peptides covering the length of three viral proteins, capsid, E2 and nsP1 proteins as well as all inactivated virus particles. Cytokine production was assessed by ELISPOT and intracellular labeling. T cells producing IFN-γ were detected against CHIKV in 85% patient’s cells either by direct ELISPOT assay (69% of patients) or after expansion of memory T cells allowing the detection of both CD4 and CD8 specific-T cells in 16% additional cases. The IFN-γ response was mainly engaged in response to nsP1 or E2 (52% and 46% cases, respectively) but in only 27% cases against the capsid. The anti-E2 response represented half the magnitude of the total CHIKV IFN-γ production and was mainly directed against the C-terminal half part of the protein. Almost all patients had conserved a T cell specific response against CHIKV with a clear hierarchy of T cell responses (CD8 > CD4) engaged against E2 > nsP1 > capsid. More importantly, the intensity of responses was not significantly different between recovered and chronic patients. These findings constitute key elements to a better understanding of patient T cell immunoreactivity against CHIKV and argue against a possible defect of T cell immunoresponse in the chronicity post-CHIKV infection.

## Introduction

Chikungunya virus (CHIKV) is a small enveloped alphavirus of the *Togaviridae* family. Like other alphaviruses, it is continuously maintained in nature by transmission cycles between mosquito’s vectors and vertebrate hosts including humans [1,2]. Isolated for the first time in Tanzania in 1953, CHIKV led to several outbreaks in Africa and Asia and has affected more than 3 million people in the Indian ocean zone, reaching Europe in 2005-2007 [3-7]. In 2005-2006, 266.000 clinical cases (about 1/3^rd^ of the population) were reported in La Reunion Island, revealing exceptional forms of the CHIKV disease (CHIKVD) in a non-immune population, including severe complications in adults (persistent arthralgia, arthritis, neurological complications ), encephalitis in newborns and increasing human morbidity [8-10].

After 2-4 days of infection, the acute phase of CHIKVD symptoms is characterized by a sudden appearance of high fever, skin rash and painful arthralgia (>90% of cases) during 3-7 days, associated or not with other symptoms like myalgia, headache, edema or gastrointestinal disorders. Like other alphaviral arthritides, such as Ross River virus disease, rheumatic manifestations can persist in a fraction (10-20%) of CHIKV patients for several weeks, months or even years. During the chronic phase, joint pains affecting wrists, elbows, toes, ankles and knees appears in a fluctuating manner but without changing anatomical location [2,11-13]. 

Several cases of post-CHIKV rheumatoid arthritis (RA)-like illnesses have been reported, with the persistence of CHIKV IgM possibly linked to the sanctuarization of the virus as observed in synovial macrophages in chronic patients but without the classical inflammation and erosion of the cartilage and bone observed in autoimmune RA [12,14].

To eradicate a viral infection, several pathways involved in antiviral defense should be coordinated. Any defect in this protective and immune mechanism could contribute to an inefficient antiviral response which could lead to a viral persistence and/or chronic arthralgia. Like for Sindbis virus and Eastern Equine encephalitis, interferon (type I and II) should play an essential role in the clearance of CHIKV [15-18]. As T and B lymphocytes and dendritic cells were shown not to be infected by CHIKV, only a limited number of studies have focused on the link between the adaptive immunity and the CHIKVD pathogenesis [12,18-20]. Here, we conducted an ex-vivo study on a cohort of 48 patients, infected by CHIKV during the 2005-2006 outbreak in La Reunion Island, to establish the nature of the specific T lymphocyte immune response occurring between patients who “recovered” or suffered from “chronic” arthralgia 1 to-2 years post-infection (p.i) against three CHIKV proteins (E2, capsid and nsP1).

## Materials and Methods

### Study subjects and samples processing

The study involved 48 patients infected by CHIKV (specific anti-CHIKV IgG+) during the 2005/2006 epidemic in La Reunion Island. The chronic status of the disease was established at least 12 months p.i as persisting pain with relapsing arthralgia in more than one small articulation (Table 1). The study was approved by the Tours IRB, France (Agreement 2006-10) and all patients signed an informed consent for participation. Peripheral Blood Mononuclear Cells (PBMC) from each patient were isolated by Ficoll-Histopaque (Pan Biotech, Germany) density gradient centrifugation from whole blood collected in 10 ml Vacutainers® containing ACD anticoagulants. Cells were studied within 24h of collection or cryopreserved at 5-10x10^6^ cells/ml in cold fetal calf serum (FCS, Pan Biotech) containing 10% dimethyl sulfoxide at -150°C (ULT Freezer, SANYO). When required, frozen cells were quickly thawed to 37°C and washed in RPMI 1640 (Pan Biotech) containing 20% FCS. Viability was invariably ≥85%.

**Table 1:** Clinical data of recovered and chronic CHIKV patients at the date of investigation.

		**Chronic** (n=39)		**Recovered** (n=9)	
	Standard	Mean	+/- SEM	Mean	+/- SEM
Sex ratio (F/M)		1.79	(25/14)	1.25	(5/4)
Age (Years)		55.82	+/- 2.56	52.11	+/- 5.27
Date Pi (Months)		14.43	+/- 0.78	12.81	+/- 0.72
IgM anti CHIKV (OD)		0.12	+/- 0.02	0.08	+/- 0.02
IgG anti CHIKV (OD)		1.57	+/- 0.05	1.49	+/- 0.13
Platelets (10^9^/L)	150-450	253.51	+/- 10.28	231.29	+/- 15.30
Neutrophils (10^9^/L)	1.4-7	4.30	+/- 0.31	4.31	+/- 0.63
Leucocytes (10^9^/L)	3-10	7.29	+/- 0.34	7.24	+/- 0.67
Lymphocyte (10^9^/L)	1.2-4	2.26	+/- 0.09	2.40	+/- 0.25
Monocytes (10^9^/L)*	0.1-1	0.46	+/- 0.02	0.39	+/- 0.05	p=0.06
CRP (mg/mL)	0-10	**12.03**	**+/- 3.33**	2.80	+/- 0.37
ALAT (UI/L)	7-31	17.21	+/- 1.48	18.44	+/- 3.03
ASAT (UI/L)	8-31	19.16	+/- 1.02	22.11	+/- 3.41
Lipase (UI/L)	0-60	34.27	+/- 4.01	42.00	+/- 2.79

The status of chronic or recovered patients was recorded at the time of investigation ranging between 6 and 24 months post-infection (Pi). Sex ratio, mean age and biochemical values are indicated as mean +/- SEM including physiological range values. No obvious abnormal biological parameters were observed except CRP values over physiological range in chronic patients. *even if not highly significant (p=0.06), an elevated number of monocytes in the chronic group of patients than in the recovered one was observed. OD = optical density.

### CHIKV Synthetic Peptides and UV Inactivated Virus

Synthetic peptides (20 amino acids longs with an offset of 10 amino-acids) spanning the entire E2, capsid and nsP1 molecules were synthesized with a purity >80% by Sigma-Genosys (UK). For each molecule, two pools of peptides (E2.1, 21 peptides; E2.2, 21 peptides; capsid.1, 12 peptides; capsid.2, 13 peptides; nsP1.1, 26 peptides and nsP1.2, 27 peptides) corresponding respectively to the amino-terminal half (Nt-half) and carboxyl-terminal half (Ct-half) regions were reconstituted at a stock concentration of 40 μg/ml in the appropriate solvent. Inactivated CHIKV was obtained by UV inactivation as previously described [21].

### ELISPOT assay

96-well polyvinylidene difluoride-bottom plates (Millipore, France) were coated with capture anti-human recombinant IFN-γ mAb (Diaclone, France) at 4 °C overnight. PBMC were added to triplicate wells (10^5^ cells/well) and challenge against each CHIKV pool of peptides (2 μg/ml each), 2 pool of EBV peptides (2 μg/ml each), phytohemagglutinin (PHA, 0.5 μg/ml) (Murex, France) and a negative control (medium alone) for 16 to 18 h at 37 °C in 5% CO2. After incubation, cells were removed and biotinylated anti-IFN-γ mAb (Diaclone) was added followed by streptavidin–alkaline phosphatase conjugate (Amersham, France) and chromogenic substrate (1-Step NBT/BCIP, Sigma-Aldrich, France) before washing the plates with tap water and counting the number of spot-forming cells (SFC) with a stereomicroscope (Carl Zeiss, France). Results were considered as positive if above the mean+3 SD with a cut off of 50 SFC/10^6^ PBMC above mean background.

### Lymphocyte proliferation assay (LPA) and intracellular cytokine secretion (ICS) determination

Cells were suspended at 2.10^6^ cells/ml in assay medium supplemented with 10% FCS and treated immediately for LPA or kept overnight at 37°C in 5% CO_2_ for ICS. For LPA, Interleukin 2 (IL-2) (Roche, France) was added on day 1 (20 UI/ml) with replacement of half of the medium at day 4 and 7. At day 11, cells were placed in supplemented medium without IL-2 for 2 days. For ICS, 10^6^ cells were incubated at 37°C in 5% CO_2_ and challenged for CHIKV E2, capsid or nsP1 pools of peptides (2 μg/ml) or complete UV inactivated virus (1/10 dilution), PHA (0.5 μg/ml) and a negative control (medium alone) for 1 h before addition of 5 μg/ml of Brefeldin A (Sigma) for 18 h. Cells were then fixed and stained for IL-2, gamma interferon (IFN-γ), CD4 or CD8 and isotype controls. For each condition, 100000 cells were analyzed with a Coulter XL flow cytometer (Beckman Coulter, France).

### Western Blotting to screen for anti-CHIKV immunoreactivity in patient’s sera

C6/36 cells (from ECACC) were grown in 75cm^2^ flasks and infected with CHIKV at M.O.I. of 1 and 10 for 24 hours. Cells were harvested with a scraper and resuspended in lysis buffer (1X PBS, 1% Triton X-100, and 1 mM EDTA with a cocktail of protease inhibitors, all at 1 μg/ml: PMSF, pepstatin A, leupeptin, and aprotinin). Protein extracts were added with 1 vol of loading buffer (0.1 M Tris, 10% glycerol, and 2% SDS) according to Laemmli’s protocol. 50 μg of sample was loaded onto 4–12% precast NuPAGE gels (Invitrogen). After electrophoretic migration, proteins were electrotransferred onto a nitrocellulose membrane (Millipore, Billerica, MA, USA). Membranes were incubated with patient’s sera followed by HPRO-conjugated secondary antibody (goat anti-human IgG, Sigma-Aldrich) and revealed with the Vector NovaRed detection kit (Vector Labs). The membranes were scanned and the intensity the bands corresponding to each CHIKV proteins detected was measured with Image J software v1.47.

### Statistical analysis

The 48 patient’s database is composed of variables which are most often continuous quantitative. Some were analyzed in their initial measures while others were treated after discretization in binomial variables. Due to the number of subjects and the distribution of measures carried out, non-parametric or permutation tests were performed. Associations between nominal variables were tested by chi-square test or Fisher exact test. Comparisons of quantitative measures between groups were realized by Mann-Withney U or Kruskall-Wallis test and correlations were established by the Spearman's rank test. P values > 0.05 were considered not significant (NS).

## Results

### Patient’s biological and immunological parameters at M12-M24 after CHIKV infection

At time of investigation, 39/48 patients with persisting arthralgia were considered as chronic (5 recovering 3 months post-analysis) whereas 9/48 patients for whom those symptoms had completely disappeared for at least 3 months were considered as recovered. Age and sex ratio did not differ significantly between the chronic and recovered groups (Mean age ± SEM of 55, 8±2.6 and 52.1±5.3 respectively) with 44% and 36% of men respectively (p-value (p) = 0.71; power of the test (P) <10%). Among the chronic symptoms, rheumatoid psoriasis was diagnosed in 4 patients, 4 had rheumatoid arthritis, 2 with spondylarthritis, periostic apposition in 3 and gout arthropathy in 1 patient. The other chronic patients could not be clearly classified from a rheumatological standpoint. In general and remarkably, no statistically aberrant biological parameters were observed among the cohort of patients at 12 and 24 months PI. Moreover, apart from a slight increase of CRP levels in chronic patients (12.03+/-3.33 mg/mL), no significant differences in the specific anti-CHIKV IgG and IgM immune responses between the two groups was observed (Table 1). In addition, we also tested the specificity of six chronic and six recovered patient’s sera by Western blotting of CHIKV-infected C6/36 cell lysates. Three major proteins from CHIKV were immunodetected (pE2, E1/E2 and capsid) and no significant immunoreactivity differences were observed between the recovered and chronic groups (Figure S1 and Table S1). 

### CHIKV specific T cell-mediated immunity

T cells were challenged against 6 pools of overlapping 20-mer synthetic peptides covering the entire E2, capsid and nsP1 proteins of CHIKV and 2 pools of EBV as an internal control.

**Figure 1:**
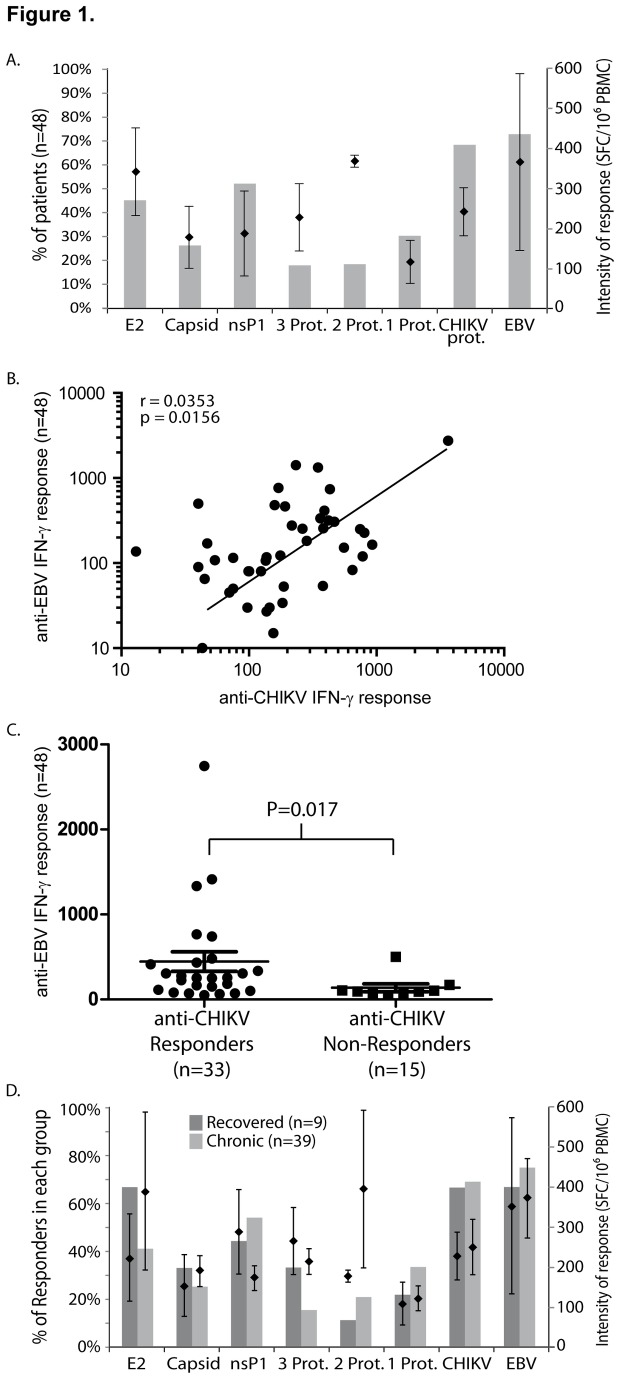
Ex vivo IFN-γ response against CHIKV E2, nsP1 or capsid and EBV. PBMCs from 48 patients were tested using an IFN-γ ELISpot after challenging against CHIKV E2, capsid or nsP1 pools of peptides or EBV. A) Profile of the T cell specific response to CHIKV compared to EBV. B) Positive correlation between the IFN-γ T cell response against CHIKV and EBV. The correlation (r) and p values are indicated. C) Anti-EBV IFN-γ T cell response between anti-CHIKV responders and Non-Responders. D) Distribution of the IFN-γ response of patients PBMC according to their clinical status. A & D) For each condition, the percentage or responders (bars) and the corresponding intensity of response against CHIKV (number of spot-forming cells (SFC)) expressed as means ± SEM per million of PBMC (dots) are represented.

Among the 48 patients, the IFN-γ producing T cell response was observed *ex vivo* against the three CHIKV proteins in 33 (69% of patients = CHIKV responders) compared to 35 (73% of patients) for EBV (p>0.05) (Figure 1A). The intensity of responses (Mean ± SEM) against the two viruses were not significantly different with respectively 243±56 and 366±89 SFC/10^6^ PBMC against CHIKV and EBV (p=0.22) and significantly correlated (r=0.0353, p=0.0156) (Figure 1B). Noteworthy, CHIKV ELISpot responders had a significantly higher response against EBV compared to CHIKV Non-Responders (p=0.017) (Figure 1C).

Fifteen of the CHIKV responders (31% of total patients) showed a response against only one CHIKV protein, with a majority responding to respectively nsP1 (8 cases), E2 (5 cases) and capsid (2 cases). Polyspecific responses were observed in 9 cases (19%) against two proteins and also in 9 other cases against the three proteins tested (Figure 1A and Table S2). Altogether, the IFN-γ response was mainly observed in response to nsP1 (n=25, 52% of patients) or E2 (n=22, 46% of patients) while capsid was driving a T cell response in only 13 cases (27%) (Figure 1A).

In terms of intensity, the response against E2 was the strongest one (342±146 SFC/10^6^ PBMC) representing half the magnitude (48±21%) of the total response against CHIKV compared to nsP1 (189±31 SFC/10^6^ PBMC representing 27±4%) while the capsid represented 25±5% of the total response with a mean of 179±33 SFC/10^6^ PBMC, thus showing clearly a hierarchy of response with E2>nsP1≥capsid (Table S2). The intensity of responses was not significantly different between recovered and chronic patients (Figure 1D).

### Characterization of multifunctional CHIKV specific T cells

In order to characterize the nature of the CD4 or CD8 T cells involved in the cytokine release in the ELISpot assay, cells from 13 of the ELISpot CHIKV-responders (Rp) were tested by ICS in an *ex-vivo* assay and evaluating the production of IFN-γ or IL-2. An IFN-γ response, mainly produced by CD8 compared to CD4 T cells (p=0.0077), was observed in all patients. In almost all cases, the IL-2 production profile was similar to what was observed for IFN-γ but at much lower frequency (and non-detected for at least 3 patients) for both CD4 (0.03±0.01%) and CD8 T cells (0.05± 0.01%) (Figure S2 & Table S3). 

### Characterization of non CHIKV-responders (NRp)

T cell responses were undetectable *ex vivo* by ELISpot in 31% of all patients. Compared to the whole study group this Non-Responder (NRp) group did not differ in age (53.5±4.3 and 56.4±2.7 years old, respectively) and whether they were recovered or chronic patients (69% and 67%, respectively), but contained twice more female than male (sex ratio of 2 versus 1.54). Of critical note, the Rp group differed from the NRp group by a significantly stronger response to control EBV antigens (446±87 for responders versus 137±46 SFC/10^6^ PBMC for non-CHIKV responders, p=0.017) suggesting some general poorer cell-mediated immunity in these NRp patients.

In order to test whether this lack of response could reflect a polarized response against other CHIKV antigens, cells from 5 NRp and 2 controls Rp were tested against the entire CHIKV inactivated by UV. An IFN-γ ELISpot response could be detected in only 2 out of the 5 NRp and yet both Rp (Table S4). This suggests that the immune response may be directed against other CHIKV proteins in these two patients and that the other three patients have no persisting or detectable anti-CHIKV T cell specific response but with a possible IgG response.

### Characterization of anti-CHIKV memory T cells

**Figure 2:**
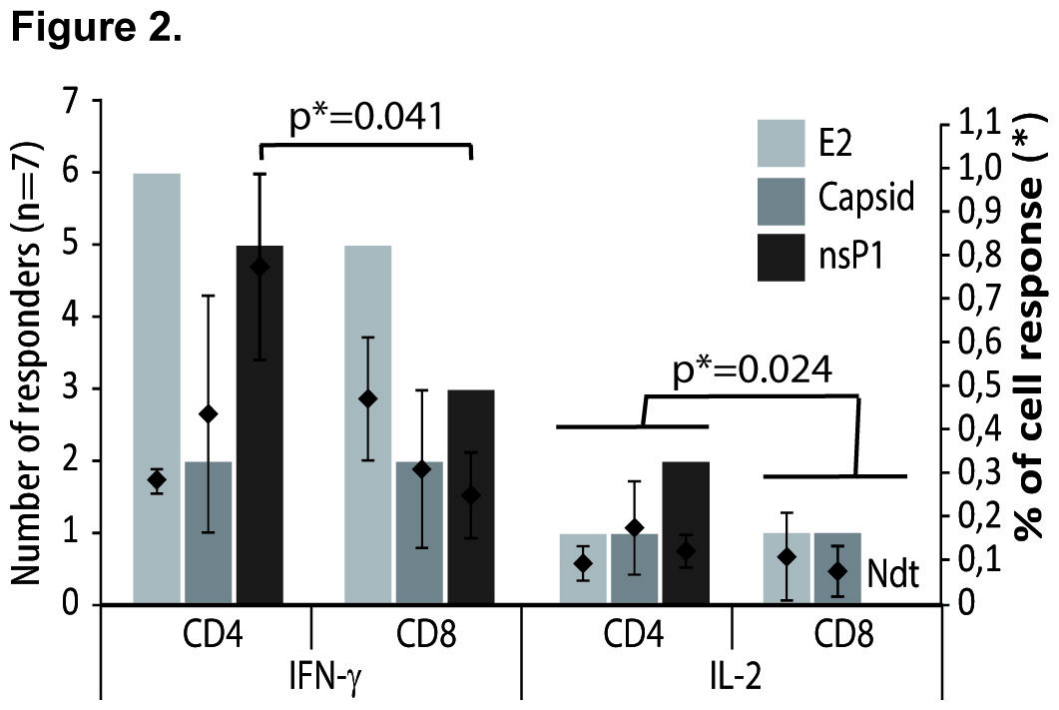
The specific CD4 versus CD8 anti-CHIKV T cell responses. After stimulation, PBMC from 7 non CHIKV-responders in ELISpot (NRp) were challenged against CHIKV E2, nsP1 or capsid pools of peptides. The reactivity of T CD4+ and T CD8+ cells producing IFN-γ or IL-2 was evaluated by intra-cellular cytokine staining and was detected in only 6 of the 7 NRps. For each condition, the number or responders (left scale) and the percentages of T cell response (means ± SEM, right scale) are indicated. Significant differences in the percentage of cell response measured using Mann–Whitney U test are indicated (p values). Ndt (Not detected).

We then evaluated the memory T cell responses in NRp after LPA. Based on the cell availability, 7 of the 15 CHIKV NRp were used. Cell reactivity was tested by intra-cellular cytokine staining and the CD4+ and CD8+ T cell origin with the production of IL-2 and IFN-γ was evaluated after peptide re-stimulation. Memory T cells could be detected in 6 of the 7 patients cells that grew in culture. In both CD4+ and CD8+ T cells, the IFN-γ response was mostly directed against nsP1 and E2. This culture amplified mostly CHIKV-specific CD4 T cells. After stimulation by nsP1, the percentage of T cells producing IFN-γ was significantly higher in CD4+ than in CD8+ T cells (p=0.041). Also, the cumulative percentage of T cells producing IL-2 after separate stimulation by E2, capsid and nsP1 was significantly higher in CD4+ than in CD8+ cells (p=0.024) (Figure 2).

Therefore, altogether among the 15 NRp in the *ex vivo* peptide ELISpot assay, 9 could be tested and a response detected in 8 of them either against the inactivated virus (2/5) or after *in vitro* culture (6/7), demonstrating an overall anti-CHIKV T cell response in at least 85% of tested patients with a CHIKV immunity (41/48).

### Specific viral protein domains driving the T cell response

**Figure 3:**
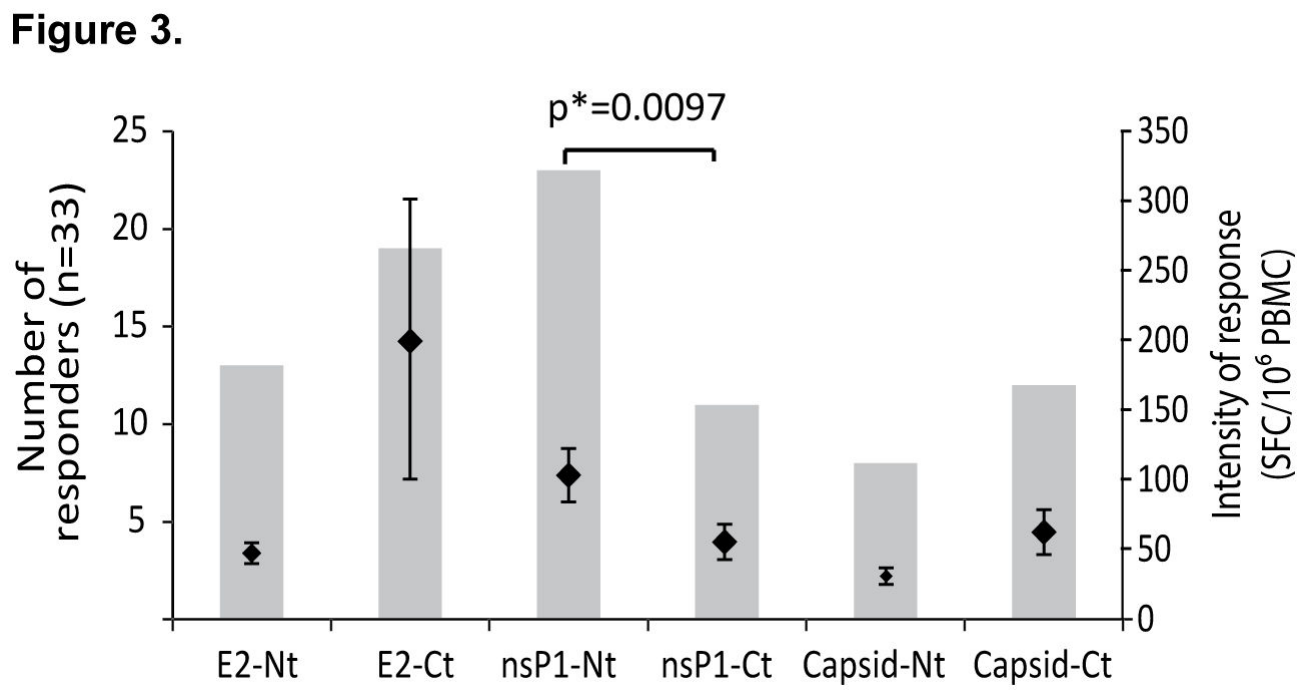
The role of the Nt and Ct domains of E2, nsP1 and capsid in the T cell responses. The response of patients PBMC following stimulation by the pools of peptides covering the amino terminal half (Nt) or the carboxy terminal half (Ct) of the three CHIKV proteins (E2, nsP1 and capsid) was measured by an IFN-γ ELISpot assay. For each condition, the number or responders (left scale, grey bars) and the intensity of response expressed as mean ± SEM of spot-forming cells (SFC) per million of PBMC (right scale, black whiskers) are represented. Significant differences in the intensity of IFN-γ response measured using Mann–Whitney U test are indicated (p values).

The regions of the three proteins targeting those responses were analyzed by using distinct pools of peptides covering the Nt-half or Ct-half of each protein. Among the 33 anti-CHIKV Rp in ELISpot, the dominant response against E2 was mainly directed against the Ct-half part in 58% of cases versus 39% responses against the Nt-half with corresponding intensity of response of 201±101 and 47±7 SFC/10^6^ PBMC (p= 0,084, P <10%). In addition, the response against the Ct-half of the capsid was also more prominent (36% of response with an intensity of 62±16 SFC/10^6^ PBMC) versus 24% responses against the Nt-half with an intensity of 30±6 SFC/10^6^ PBMC (p=0.76, P<10%). Finally, the difference was more pronounced against nsP1 with 70% of the patients responding against the Nt-half versus twofold less (33%) against the Ct-half with respective intensities of response of 103±19 and 55±13 SFC/10^6^ PBMC (p=0.0097). All together, these results suggest that the Ct- half of the E2 contains key antigens for the T cell responses against CHIKV, while the Nt-half is dominant for nsP1 (Figure 3).

### Possible link between the clinical status of the patients and the pattern of CHIKV-specific T cell responses at 12-24 months p.i ?

When analyzing antigen recognition according to the clinical status (chronic vs. recovered) of the patients, no significant differences were observed in the IFN-γ producing responses for both the relative frequency and the intensity of response against EBV or CHIKV. Among the recovered patients (n=9), E2 was the most frequently recognized antigen (6 patients, 67 %) followed by nsP1 (4 patients, 44%) and capsid (3 patients, 33%). In contrast, in chronic patients (n=39), nsP1 was slightly dominant (21 patients, 54 %) followed by E2 (16 patients, 41%) while the capsid was a minor component of the immune response (10 patients, 26%). Interestingly, among the 33 anti-CHIKV responders in ELISpot, all recovered patients (6/6) were anti-E2 responders whereas only 16 out of the 27 chronic patients were E2 responders suggesting that E2 could play an essential role in the process of complete recovery. However, for the three CHIKV proteins tested, no significant difference of intensity in the ELISpot responses between recovered and chronic was evidenced (Figure 1D). 

## Discussion

The clinical follow up of CHIKV-infected patients has revealed that an estimated 10-20% of the patients have developed persistent chronic manifestations [10,12,22] and yet little was known about the T cell reactivity in both recovered and chronic patients. 

Following the CHIKV epidemic in La Reunion Island in 2005-2006 in a non-immune population, we investigated the involvement of CHIKV specific T cells in either “recovered” or “chronic” group of patients. In our cohort of 48 patients, clinical biological laboratory parameters remained largely within the normal ranges except for a slightly elevated CRP in the chronic group underlining the presence of a mild chronic inflammatory process. Of note, some patients presented arthritogenic symptoms (29% of patients) necessitating treatment with methotrexate [23]. The IgG and IgM levels were not significantly different between the two groups whereas in a cohort of Indian patients with post-CHIKV rheumatoid arthritis-like illnesses, Chopra et al. [14] reported high levels of CHIKV IgM. Moreover, no significant difference of age was evidenced between chronic and recovered patients even if clinical cohort studies have reported that age is a critical factor linked to more severe CHIKV pathologies (at least in the acute phase of the pathology) and subsequent sequelae [8,24,25]. 

For each patient, and due to the limitation of PBMCs samples, we restricted the study to the capacity of T cells to respond to two major immunogenic CHIKV structural proteins (E2 and capsid, as evidenced by Western-blot analysis) compared to a single non-structural protein (nsP1). We also screened for the specificity of the anti-CHIKV antibodies in our patient sera by Western blotting of CHIKV-infected C6/36 cell lysates and found that the humoral IgG immune response directed against E1/E2 and capsid [12] was equivalent between chronic and recovered patients (n=6).

At the time of investigation (>12months p.i), the majority but not all of the patients had conserved T cells able to induce a detectable IFN-γ production in ELISpot against the three CHIKV proteins studied (E2, nsP1 and capsid). E2 and nsP1 were the most frequently targeted proteins but the T cell IFN-γ intensity response was mainly induced by E2. As a consequence, E2 envelop protein seems to play a more prominent role in the anti-CHIKV T cell mediated immunity than nsP1, which is produced during the replication phase of the virus. 

In contrast, CHIKV T cell responses against the pools of capsid peptides were less frequent and of weaker intensities when compared to the T cell IFN-γ response engaged by E2 and nsP1 peptides. This could lead, in the context of MHC presentation and/or recognition by T cells of capsid antigenic peptides, to a defect in the cellular immunity driven by CD8+ T cells or a default in the orchestration of the immune response directed by CD4+ helper T-cells.

When comparing the T cell response depending on the clinical status of the patients, E2 envelop protein was more frequently targeted in the recovered group compared to nsP1 in the chronic group, indicating that E2 could play an essential role in the process of complete recovery. Moreover, the stronger intensity of the T cell IFN-γ response induced by nsP1 in the recovered group could also participate to the recovering process. 

Focusing on the NRp patients in ELISpot, we checked and confirmed that this group had a conserved memory T cells but necessitating a phase of proliferation to produce an efficient IFN-γ and/or IL-2 response. During the first days of the acute phase of the infection, a strong activation of the innate immune response involving the production of antiviral IFN type I, pro-inflammatory cytokines, chemokine’s and cytokines has been largely described. More recently, an up-regulation of activated CD8+ T cells contributing to the elevated level of IFN-γ during the acute phase of CHIKV infection was seen to be associated with a down-regulation of CD4+ T cells probably due to an induced early apoptosis through CD95/CD95L interaction [12,19]. At the late stage of the acute phase, Wauquier et al. described a classical switch to CD4+ T cells response with the production of anti-inflammatory IL-1rα and IL-2RA [19]. Interestingly, our results indicate that during the chronic phase of the disease, the IFN-γ T cell response was mainly driven by CD8+ T cells except in a minority of patient’s necessitating a prior T lymphocyte proliferation and which led to a response mainly engaged by CD4+ T cells.

Interestingly, the intensity of IFN-γ response against EBV antigens was significantly stronger in the group of “CHIKV Rp” compared to “CHIKV NRp” by ELISpot. Together, this indicated a general poorer cell-mediated immunity in the NRp which could explain a less propensity to mount a stable anti-CHIKV immunity [26].

As already described for other alphaviruses, the identification of immunogenic regions of each viral proteins targeted by T cells but also B cells is essential [27-29]. Our results have clearly identified, among the three CHIKV proteins studied, E2 envelop protein of CHIKV as a major target of T cells. Recently, the crystal structure of CHIKV E1-E2 glycoprotein was resolved and allowed to identify the N-terminal part of E2 as a prominently exposed region on the surface of the virus which was found to be targeted by antibodies of the IgG3 isotype [20,30,31]. Here, we demonstrated that the Ct-half part of E2 protein is a key element in the anti-CHIKV T cell response inducing a high frequency of T cell response and the highest level of IFN-γ release. This immunogenicity could have been facilitated by the high viral load observed during the acute phase of the disease which could initially induce a robust immune response, probably responsible for the limiting duration of the infection, leading to the persistence of a high percentage of specific memory T cells after a long period post infection [32]. Taken together, the Ct-half of E2 and the Nt-half of nsP1 contain key elements to take in consideration for a future vaccine to drive a prominent T cell response.

Recently, Messaoudi et al (2013) compared the specific T cell response against the 9 CHIKV proteins (nsP1-4, capsid, E1-E3 and 6K) and shown a similar hierarchy of the response in aged rhesus Macaque for the 3 proteins studied herein with nsP1E2capsid whereas in adult macaque the hierarchy was slightly modified with nsP1CapsidE2 [33]. Despite the limited number of animals included in this study, advanced aged macaques presented a reduced frequency and an altered breadth of anti-CHIKV T cells response. Our results also indicate that even if almost all patients had maintained a T cell anti-CHIKV immunity, 20% had only conserved memory T cells at 1 to 2 years pi, probably linked to the fact that some of them were affected by a general poorer cell-mediated immunity.

All together, these results will participate to a better understanding of the T cell immunoreactivity against CHIKV and other alphaviruses. As the development of a vaccine constitute an essential step to protect the population of countries affected or threatened by CHIKV, these observations will need to be completed by the precise identification of common dominant epitopes able to induce both a strong T cell but also B cell efficient immune response to ensure a long lasting protection against CHIKV. In particular, other CHIKV proteins like the glycoprotein E1 which contains more conserved (non-neutralizing), cross-reactive epitopes [33-35] will need to be included in future studies as they should also contain key immunogenic epitopes targeted by T cells.

## Supporting Information

Table S1 **Comparison of the Anti-CHIKV IgG and IgM responses of 6 chronic and 6 recovered patients.** Sex ratio, mean age, IgG and IgM levels of the patients which sera were analyzed in Figure S1 were not significantly different between 9 and 18 months post-infection.
(DOCX)

Table S2 **IFN-γ ELISpot responses of CHIKV patients (n=48).** Following stimulation by the 3 pools of CHIKV or EBV peptides, the number of responders and the intensity of the T cell IFN-γ response expressed by the number of spot forming cell for 10^6^ PBMC are indicated.
(DOCX)

Table S3 **Predominance of the IFN-γ production in CD8+ T cells.** The percentage of T CD4+ and T CD8+ cells producing IFN-γ or IL-2 following CHIKV pool of peptides challenge was assessed within a multifunctional analysis for the 13 patients shown in Figure S1 indicating a clear predominance of the IFN-γ production in CD8+ T cells.
(DOCX)

Table S4 **IFN-γ response of Non-responders (NRp) against inactivated CHIKV.** PBMCs from 7 patients (5 NRp and 2 Rp) were challenged against complete CHIKV inactivated by UV or EBV. A number of SFC/10^6^ cells (*) ≥ 50 was considered as a positive response.
(DOCX)

Figure S1 **Specificity of the anti-CHIKV antibodies of patient serums.** Western blot analyses were performed to determine the CHIKV proteins identified by patient’s serum. A) Three main CHIKV proteins: Pre-E2 (pE2), E1 and E2 (E1/E2) and capsid were detected by anti-CHIKV antibodies contained in the tested serums (6 were from chronic and 6 from recovered patients). B) The evaluation of the intensity of each CHIKV proteins detected in the different patient’s serum revealed no significant difference in the immunoreactivity between the two groups (p>0.05). For each patient, the serum immunoreactivity was evaluated against Mock-infected C6/36 cells (Lane 1) and CHIKV-infected C6/36 cells at MOI of 10 (Lane 2) and MOI of 1 (Lane 3). Arrows on the right indicate the CHIKV proteins detected by Western blot. A protein size marker is shown on the left. Each Patient number is indicated below the gels.
(TIF)

Figure S2 **CD4+ and CD8+ T cells involved in the anti-CHIKV response.** T cells from 13 patients responding in ELISpot were challenged again with appropriate CHIKV pools of peptides to determine the percentage of CD8+ and CD4+ cells producing IFN-γ or IL-2. Wiskers plots (Sigma Plot) represent the distribution of the T cells percentage for the 13 patients.
(TIF)
