# Supplementary material for: Identical Strength of the T Cell Responses against E2, nsP1 and Capsid CHIKV Proteins in Recovered and Chronic Patients after the Epidemics of 2005-2006 in La Reunion Island
Source: PLoS One. 2013 Dec 23;8(12):e84695. doi: 10.1371/journal.pone.0084695 (PMC3871564; doi:10.1371/journal.pone.0084695)
Supplement: Table S1 — Comparison of the Anti-CHIKV IgG and IgM responses of 6 chronic and 6 recovered patients. Sex ratio, mean age, IgG and IgM levels of the patients which sera were analyzed in Figure S1 were not significantly different between 9 and 18 months post-infection. (DOCX) [file pone.0084695.s001.docx]

**Table S1.** Identical anti-CHIKV antibody responses of six recovered and six chronic age matched patients

|  |  |  |  | Anti-CHIKV (OD) | |
| --- | --- | --- | --- | --- | --- |
| Patients | Sexe | Age | Months p.i | IgM | IgG |
| Recovered |  |  |  |  |  |
| 0001 | M | 46 | 12,1 | 0,04 | 1,00 |
| 4042 | F | 46 | 11,0 | 0,03 | 1,96 |
| 0047 | F | 61 | 13,0 | 0,19 | 1,82 |
| 1026 | F | 59 | 9,0 | 0,03 | 1,51 |
| 4019 | F | 31 | 14,1 | 0,05 | 1,76 |
| 1018 | M | 56 | 9,1 | 0,28 | 1,65 |
| Mean |  | 50 | 11 | 0,10 | 1,62 |
| SD |  | 9 | 2 | 0,09 | 0,29 |
|  |  |  |  |  |  |
| Chronic |  |  |  |  |  |
| 1073 | M | 38 | 18,7 | 0,05 | 1,92 |
| 0032 | F | 58 | 11,2 | 0,06 | 1,57 |
| 1076 | M | 53 | 17,1 | 0,07 | 2,00 |
| 1061 | M | 54 | 15,6 | 0,05 | 1,37 |
| 1051 | M | 43 | 10,2 | 0,38 | 1,89 |
| 1019 | M | 74 | 8,8 | 0,14 | 1,64 |
| Mean |  | 53 | 14 | 0,12 | 1,73 |
| SD |  | 11 | 3 | 0,11 | 0,21 |
|  |  |  |  |  |  |
| *p value* | 0,24 | 0,94 | 0,48 | 0,29 | 0,70 |
