# Supplementary material for: Identical Strength of the T Cell Responses against E2, nsP1 and Capsid CHIKV Proteins in Recovered and Chronic Patients after the Epidemics of 2005-2006 in La Reunion Island
Source: PLoS One. 2013 Dec 23;8(12):e84695. doi: 10.1371/journal.pone.0084695 (PMC3871564; doi:10.1371/journal.pone.0084695)
Supplement: Table S2 — IFN-γ ELISpot responses of CHIKV patients (n=48). Following stimulation by the 3 pools of CHIKV or EBV peptides, the number of responders and the intensity of the T cell IFN-γ response expressed by the number of spot forming cell for 106 PBMC are indicated. (DOCX) [file pone.0084695.s002.docx]

**Table S2.** IFN-γ ELISpot responses of patients

|  |  |  | **Patients response** | | **IFN-γ response** (SFC/10^6^ cells) | |
| --- | --- | --- | --- | --- | --- | --- |
|  | **peptides Pools recognized** | | n= | Total % (n=48) | Mean ± | SEM |
| CHIKV | E2 | | 22 | 46% | 342.3 ± | 145.8 |
|  | nsP1 | | 25 | 52% | 188.5 ± | 31.0 |
|  | Capsid | | 13 | 27% | 179.2 ± | 33.3 |
|  | 3 pools | E2+nsP1+Capsid | 9 | 19% | 228.6 ± | 34.5 |
|  | 2 pools | E2+nsP1 | 7 | 19% | 197.5 ± | 37.2 |
|  |  | E2+Capsid | 1 |  | 1791.7 ± | 1511.7 |
|  |  | nsP1+Capsid | 1 |  | 142.5 ± | 90.5 |
|  | 1 pool | E2 | 5 | 31% | 107.0 ± | 27.0 |
|  |  | nsP1 | 8 |  | 89.3 ± | 16.5 |
|  |  | Capsid | 2 |  | 259.2 ± | 200.8 |
|  | Total responders | | 33 | 69% | 242.9 ± | 55.5 |
| EBV | Total responders | | 35 | 73% | 366.3 ± | 88.9 |
