# Supplementary material for: Identical Strength of the T Cell Responses against E2, nsP1 and Capsid CHIKV Proteins in Recovered and Chronic Patients after the Epidemics of 2005-2006 in La Reunion Island
Source: PLoS One. 2013 Dec 23;8(12):e84695. doi: 10.1371/journal.pone.0084695 (PMC3871564; doi:10.1371/journal.pone.0084695)
Supplement: Table S3 — Predominance of the IFN-γ production in CD8+ T cells. The percentage of T CD4+ and T CD8+ cells producing IFN-γ or IL-2 following CHIKV pool of peptides challenge was assessed within a multifunctional analysis for the 13 patients shown in Figure S1 indicating a clear predominance of the IFN-γ production in CD8+ T cells. (DOCX) [file pone.0084695.s003.docx]

**Table S3.** T cells response following CHIKV challenge

|  |  | **IFN-γ** | | **IL-2** | |
| --- | --- | --- | --- | --- | --- |
| **Patients** | **Stimulation** | **CD4** | **CD8** | **CD4** | **CD8** |
| 0001 | E2.2+nsP1.1 | 0.040 | 0.100 | 0.020 | 0.040 |
| 0004 | nsP1 | 0.090 | 0.180 | 0.000 | 0.010 |
| 0015 | E2.2+nsP1.1 | 0.000 | 0.240 | 0.020 | 0.000 |
| 0019 | E2.2+nsP1.1 | 0.021 | 0.220 | 0.000 | 0.000 |
| 1004 | nsP1+E2 | 0.081 | 0.170 | 0.104 | 0.050 |
| 1010 | nsP1.2+Caps.2+E2.2 | 0.011 | 0.008 | 0.086 | 0.190 |
| 1018 | Capsid.2 | 0.000 | 0.090 | 0.040 | 0.060 |
| 1019 | E2.2 | 0.070 | 0.970 | 0.000 | 0.020 |
| 1046 | nsP1 | 0.060 | 0.110 | 0.040 | 0.110 |
| 1061 | Capsid.2 | 0.050 | 0.003 | 0.000 | 0.000 |
| 1073 | E2.2 | 0.000 | 0.920 | 0.020 | 0.090 |
| 4019 | nsP1+E2 | 0.049 | 0.067 | 0.077 | 0.029 |
| 4042 | E2.2 | 0.483 | 0.257 | 0.000 | 0.000 |
