# Supplementary material for: Identical Strength of the T Cell Responses against E2, nsP1 and Capsid CHIKV Proteins in Recovered and Chronic Patients after the Epidemics of 2005-2006 in La Reunion Island
Source: PLoS One. 2013 Dec 23;8(12):e84695. doi: 10.1371/journal.pone.0084695 (PMC3871564; doi:10.1371/journal.pone.0084695)
Supplement: Table S4 — IFN-γ response of Non-responders (NRp) against inactivated CHIKV. PBMCs from 7 patients (5 NRp and 2 Rp) were challenged against complete CHIKV inactivated by UV or EBV. A number of SFC/106 cells (*) ≥ 50 was considered as a positive response. (DOCX) [file pone.0084695.s004.docx]

**Table S4.** T cells response following challenge against entire CHIKV particles inactivated by UV

|  |  | **Patients (Clinical status)** | | | | | | |
| --- | --- | --- | --- | --- | --- | --- | --- | --- |
|  |  | 1049  Chronic | 1052  Chronic | 1062  Chronic | 1066  Chronic | 1069  Chronic | 0050  Chronic | 1016  Recovered |
| **IFN-γ response (SFC/10^6^ cells) against :** | **CHIKV pool of peptides** | 400 (Rp) | 293  (Rp) | <50  (NRp) | <50  (NRp) | <50  (NRp) | <50  (NRp) | <50  (NRp) |
|  | **CHIKV inactivated by UV** | 144 | 76 | 134 | <50 | <50 | <50 | 86 |
|  | **EBV** | 414 | 166 | <50 | 52 | 108 | <50 | <50 |
