# Supplementary figures and images for: Identical Strength of the T Cell Responses against E2, nsP1 and Capsid CHIKV Proteins in Recovered and Chronic Patients after the Epidemics of 2005-2006 in La Reunion Island
Source: PLoS One. 2013 Dec 23;8(12):e84695. doi: 10.1371/journal.pone.0084695 (PMC3871564; doi:10.1371/journal.pone.0084695)

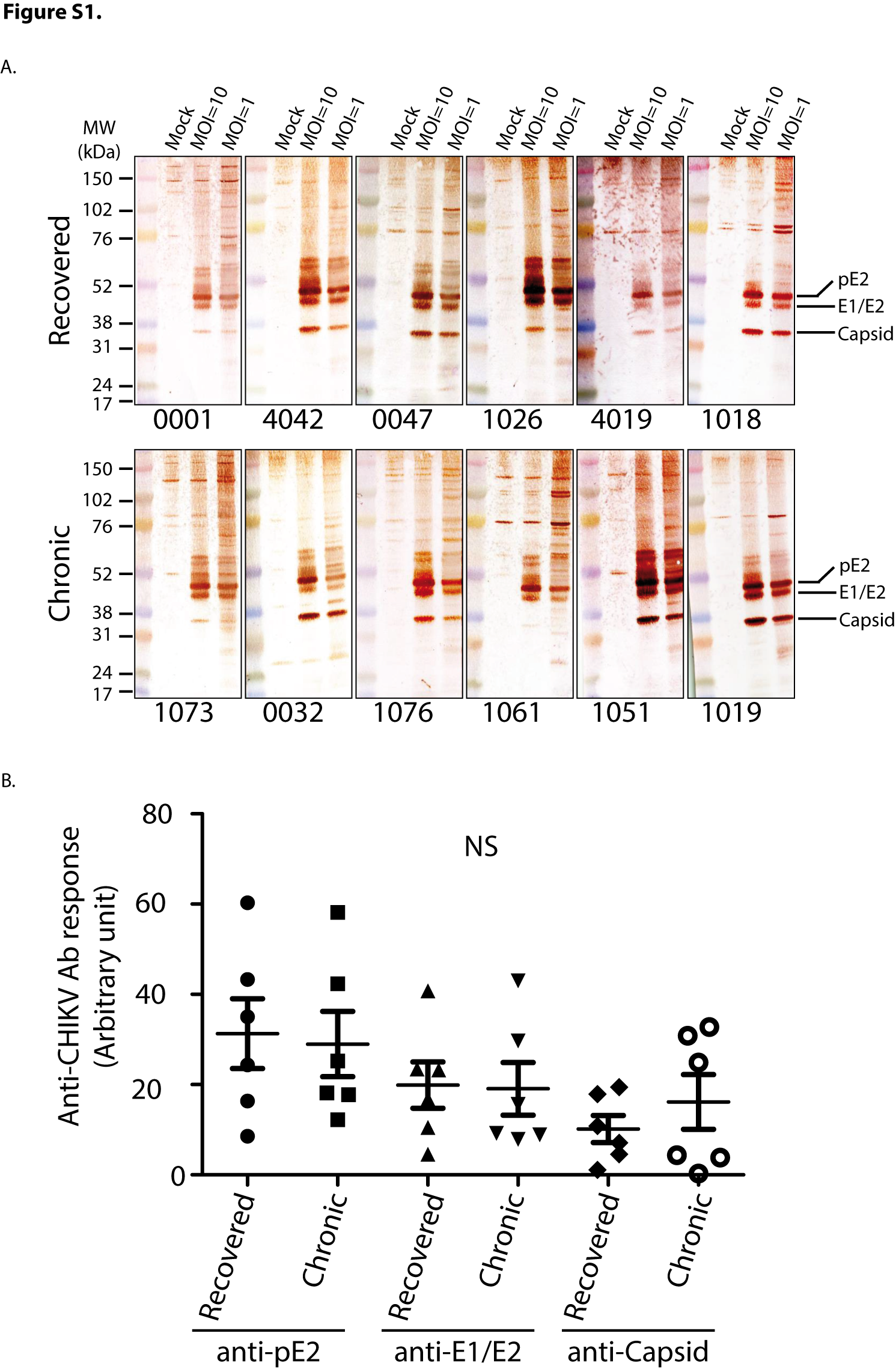

Supplement: Figure S1 — Specificity of the anti-CHIKV antibodies of patient serums. Western blot analyses were performed to determine the CHIKV proteins identified by patient’s serum. A) Three main CHIKV proteins: Pre-E2 (pE2), E1 and E2 (E1/E2) and capsid were detected by anti-CHIKV antibodies contained in the tested serums (6 were from chronic and 6 from recovered patients). B) The evaluation of the intensity of each CHIKV proteins detected in the different patient’s serum revealed no significant difference in the immunoreactivity between the two groups (p>0.05). For each patient, the serum immunoreactivity was evaluated against Mock-infected C6/36 cells (Lane 1) and CHIKV-infected C6/36 cells at MOI of 10 (Lane 2) and MOI of 1 (Lane 3). Arrows on the right indicate the CHIKV proteins detected by Western blot. A protein size marker is shown on the left. Each Patient number is indicated below the gels. (TIF) [file pone.0084695.s005.tif]

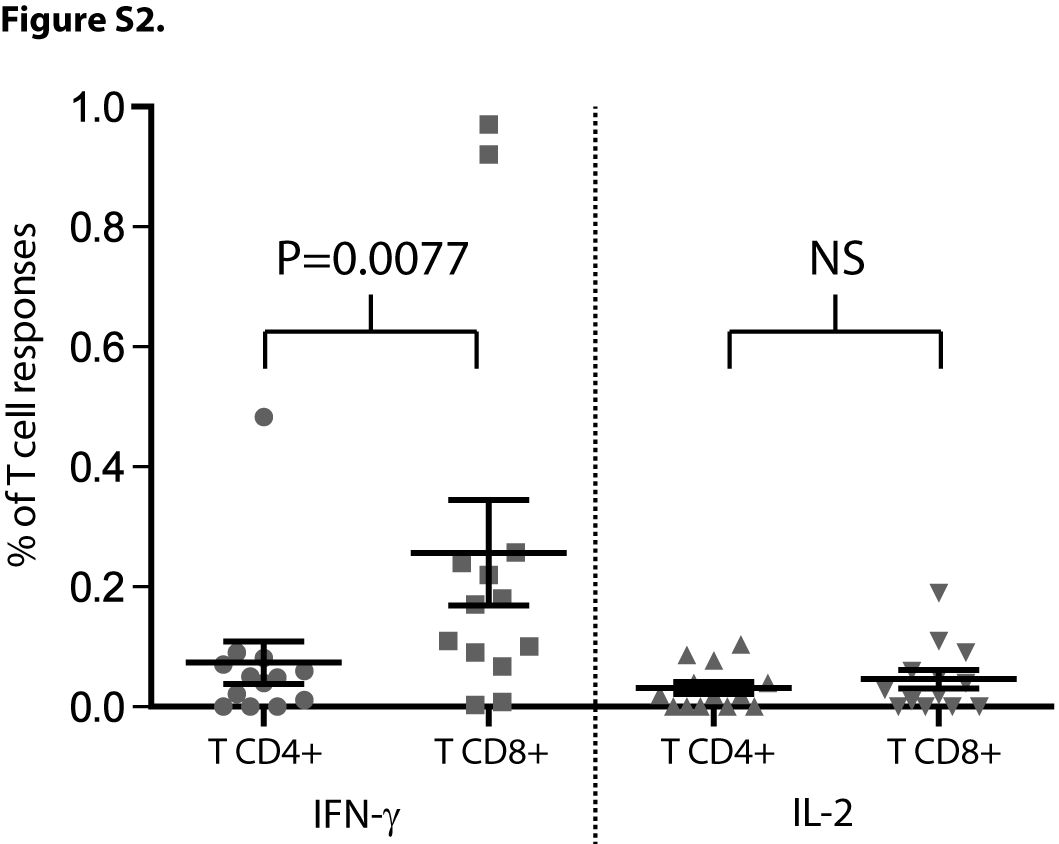

Supplement: Figure S2 — CD4+ and CD8+ T cells involved in the anti-CHIKV response. T cells from 13 patients responding in ELISpot were challenged again with appropriate CHIKV pools of peptides to determine the percentage of CD8+ and CD4+ cells producing IFN-γ or IL-2. Wiskers plots (Sigma Plot) represent the distribution of the T cells percentage for the 13 patients. (TIF) [file pone.0084695.s006.tif]
